# Ocular gene therapy as a sustained drug delivery system: pharmacokinetic and genokinetic perspectives

**DOI:** 10.25122/jml-2025-0180

**Published:** 2025-11

**Authors:** Carmen-Ecaterina Leferman, Alin Dumitru Ciubotaru

**Affiliations:** 1 Department of Pharmacology, Grigore T. Popa University of Medicine and Pharmacy, Iasi, Romania; 2 Department of Biochemistry, Grigore T. Popa University of Medicine and Pharmacy, Iasi, Romania

**Keywords:** ocular gene therapy, pharmacokinetics, sustained drug delivery, adeno-associated virus, anti-VEGF therapy, retinal diseases

## Abstract

Ocular pharmacotherapy is constrained by compartmental anatomy and clearance barriers that limit sustained posterior-segment exposure. Intravitreal bolus dosing, therefore, remains dominant for retinal disease but produces peak-trough profiles and frequent retreatment. Long-acting implants and refillable systems can prolong exposure, yet are finite or maintenance-dependent. Ocular gene therapy introduces a different paradigm in which transduced retinal cells act as localized 'biofactories,' enabling prolonged intraocular production of therapeutic proteins after a single or infrequent administration. This review integrates pharmacokinetic principles with determinants of transgene expression, including vector/capsid design, promoter architecture, route-dependent biodistribution (subretinal, intravitreal, suprachoroidal), and immune modulation, to explain typical kinetics (lag phase, rise to plateau, and potential attenuation). We highlight an infusion-equivalent modeling framework that treats transgene-driven protein output as sustained input balanced by first-order loss, providing parameters for time to plateau, steady-state exposure, and variability. Finally, we discuss translational implications for efficacy and safety, including exposure-response and therapeutic window definition in emerging retinal gene therapy programs (notably anti-VEGF), and future directions such as tunable expression systems and biomarker-linked, model-informed dose optimization.

## Introduction

Ocular pharmacotherapy has advanced substantially over the past two decades in response to the distinctive pharmacokinetic (PK) constraints of the eye [1]. Anatomical and physiological barriers – including the cornea, conjunctiva, sclera, and the blood-retina barrier – limit penetration to intraocular tissues and contribute to rapid drug loss from accessible compartments, complicating sustained management of chronic disorders such as age-related macular degeneration (AMD), diabetic retinopathy (DR), and glaucoma, particularly when therapeutic targets reside in the posterior segment [2,3]. As a result, intravitreal administration remains the dominant strategy for achieving therapeutically relevant retinal exposure. However, macromolecules administered intravitreally typically exhibit first-order decay and clinically meaningful peak–trough profiles, which translate into frequent retreatment and cumulative procedural burden over long treatment horizons [4,5].

Several strategies have been developed to reduce injection frequency, including biodegradable and non-biodegradable corticosteroid implants and refillable delivery platforms [6-8]. The Port Delivery System with ranibizumab (Susvimo) represents an important step toward near-continuous intraocular delivery and illustrates the trade-off between durability and device-level safety that necessitates long-term surveillance [8,9]. Following a voluntary recall and subsequent redesign of components, Susvimo was reintroduced in the United States in July 2024 after the Food and Drug Administration (FDA) approved updates to the implant and refill needles [10]. Even with such advances, device-based approaches remain finite systems that require maintenance, refills, or replacement.

Gene therapy offers a distinct therapeutic model by enabling localized, sustained production of therapeutic proteins after a single administration [11,12]. Rather than repeatedly delivering pharmacologically active molecules, gene-based approaches provide genetic instructions that allow ocular cells to synthesize therapeutic agents endogenously [12,13]. Adeno-associated virus (AAV) vectors can support durable expression in retinal pigment epithelium (RPE), photoreceptors, and other retinal cell populations, and clinical experience with voretigene neparvovec has established the eye as a favorable compartment for sustained transgene-driven therapy with multi-year functional benefit in human follow-up [14-16].

Building on this foundation, anti-vascular endothelial growth factor (anti-VEGF) gene therapy programs aim to reduce or eliminate chronic injection schedules in neovascular AMD (nAMD) [17]. Late-stage development includes ABBV-RGX-314 (sura-vec; NAV^®^ AAV8 encoding an anti-VEGF Fab), which is being investigated as a potential one-time treatment for nAMD and other chronic retinal conditions [18,19], and ixoberogene soroparvovec (ixo-vec; formerly ADVM-022; AAV.7m8-aflibercept), which entered the ARTEMIS Phase 3 program in 2025 [20,21].

A consistent quantitative interpretation of intraocular exposure to transgene-derived proteins remains an important translational need [22,23]. A PK framing can align determinants of expression — capsid tropism, promoter architecture, route of administration, and immune modulation — with clinically observed durability and variability [3,24,25]. In this context, the rate of gene-driven protein synthesis (*k*_expr_) can be treated as a functional analogue of a constant-rate input, while protein degradation or clearance (*k*_deg_) governs the time to plateau and the steady-state concentration [23,26]. This operational approach supports the emerging concept of ocular 'biofactories,' in which transduced cells act as endogenous producers of therapeutic proteins within a definable exposure–response framework [22,27].

In this article, we provide a narrative review at the intersection of pharmacokinetics, molecular ophthalmology, and retinal therapeutics, informed by targeted searches of major biomedical sources and key reference lists. We summarize key limitations of conventional ocular drug delivery, examine kinetic determinants of gene-based approaches, and discuss how an infusion-equivalent modeling perspective may help interpret durability, interindividual variability, and safety in emerging ocular gene therapy platforms.

## Pharmacokinetics of conventional ocular drug delivery

### Anatomical and physiological barriers

The eye is among the most pharmacokinetically challenging organs because of its compartmentalized anatomy and multiple diffusion and clearance barriers [3]. The corneal epithelium limits penetration of hydrophilic compounds, while the conjunctiva and sclera restrict the diffusion of macromolecules [1,3]. Systemic access is further constrained by the blood–aqueous and blood–retina barriers, which tightly regulate intraocular exposure [1,3].

Elimination occurs mainly through aqueous humor outflow in the anterior segment and via posterior pathways across/through the retina–choroid complex [1,3]. Together with the small vitreous volume (approximately 4 mL in adults) and limited lymphatic contribution, these pathways lead to short intraocular residence times [28,29]. Small molecules often have vitreous half-lives on the order of hours, whereas large biologics typically exhibit longer intraocular persistence on the order of days to weeks, limiting durable exposure with conventional dosing [3,4].

### Routes of administration and pharmacokinetic profiles

**Figure 1:**
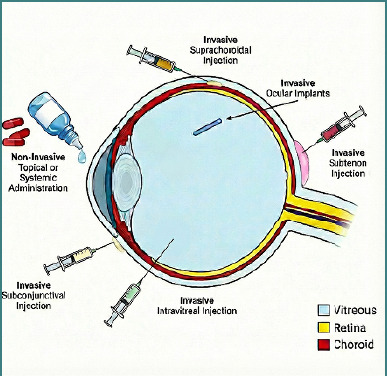
Ocular drug delivery routes and targeted intraocular compartments. Schematic overview of common routes for ocular therapy, including non-invasive topical or systemic administration and invasive local approaches (subconjunctival, sub-Tenon, intravitreal, and suprachoroidal injection) as well as ocular implants. The relative anatomical targets and compartments involved—vitreous, retina, and choroid—are indicated for each delivery route.

The PK of ocular drugs depends strongly on the route of administration (Table 1), which determines the primary site of exposure (Figure 1) and the dominant elimination pathway [1,3].

**Table 1:** Ocular routes of administration and their dominant pharmacokinetic characteristics

Route	Primary target	Representative uses	Dominant PK features / limitations
**Topical**	Anterior segment	Anterior inflammation, infection	Rapid clearance, negligible vitreoretinal exposure
**Periocular**	Sclera/uvea (regional)	Regional/posterior inflammation	Depot + trans-scleral diffusion; variable retinal delivery
**Intravitreal**	Vitreous/retina	nAMD, DME, RVO	High vitreous exposure, first-order decay; injection-related risks
**Suprachoroidal**	Choroid/outer retina	Uveitis, macular edema, gene delivery	Posterior-biased distribution, reduced anterior/systemic spillover; technique-dependent
**Subretinal**	RPE/photoreceptors (focal)	IRDs, gene therapy	High local transduction, focal exposure; surgical delivery required

nAMD, neovascular age-related macular degeneration; DME, diabetic macular edema; RVO, retinal vein occlusion; RPE, retinal pigment epithelium; IRDs, inherited retinal diseases; PK, pharmacokinetic(s)

Topical formulations remain the first-line therapy for anterior segment diseases [3,30]. Rapid tear turnover, nasolacrimal drainage, and corneal barrier function result in short precorneal residence and minimal posterior segment bioavailability, generally insufficient for chronic retinal indications [3,30].

Periocular injections (subconjunctival, sub-Tenon’s, or peribulbar) can increase exposure to the sclera and uveal tract. However, diffusion and clearance barriers often prevent therapeutic concentrations from reaching the retina or choroid. Sustained-release depots may extend local anti-inflammatory effects but provide inconsistent long-term posterior segment coverage for many retinal disorders [1,3].

Intravitreal injection bypasses most external barriers and remains the dominant route for posterior segment pharmacotherapy [1,3]. Following administration, drug concentration typically declines in a manner consistent with one-compartment, first-order elimination, necessitating repeated dosing [31,32]. Anti-VEGF biologics such as ranibizumab, aflibercept, and bevacizumab have vitreous half-lives on the order of several days (first-order decline/modeling), supporting common 4–8 week dosing schedules in clinical practice [4].

Suprachoroidal delivery targets the potential space between the sclera and choroid and enables more localized posterior distribution with reduced anterior segment exposure [33,34]. This approach may improve bioavailability to retinal and choroidal tissues and has gained interest for macular edema, uveitis, and as a potential route for gene vector delivery [33,35].

Subretinal injection places therapeutic material between the photoreceptor layer and the retinal pigment epithelium, offering direct access to key cellular targets. While surgically demanding, this route is central to many ocular gene therapy programs and supports high local transduction with limited systemic exposure [1,3].

### Controlled and sustained-release formulations

To reduce intravitreal injection frequency and stabilize intraocular exposure in chronic retinal disease, multiple controlled and sustained-release systems have been developed (Table 2) [1,3].

**Table 2:** Selected long-acting intraocular delivery systems and their pharmacokinetic properties

Therapy device	Platform	Indication (example)	Duration / PK	Key safety notes
Ozurdex^®^	Biodegradable intravitreal implant	ME (RVO), posterior uveitis	~4–6 mo; burst → decline	IOP ↑, cataract
Iluvien^®^ / Yutiq^®^	Non-biodegradable intravitreal implant	Chronic DME, uveitis	Up to ~36 mo; near zero-order	High IOP, cataract
Susvimo^®^ (PDS)	Refillable scleral reservoir	nAMD	~4–6 mo/refill; diffusion-driven	Device/surgical risks
Injectable depots (exp.)	Biodegradable depots	Investigational	Weeks–months; extended release	Long-term safety TBD

ME, macular edema; RVO, retinal vein occlusion; BRVO, branch retinal vein occlusion; CRVO, central retinal vein occlusion; DME, diabetic macular edema; nAMD, neovascular age-related macular degeneration; IOP, intraocular pressure; PDS, Port Delivery System; TBD, to be determined; exp., experimental; mo, months; PK, pharmacokinetic(s)

The dexamethasone intravitreal implant (Ozurdex^®^) is a biodegradable poly(lactic-co-glycolic acid)-based (PLGA-based) system designed to provide corticosteroid release for approximately 4–6 months and is approved for diabetic macular edema, macular edema secondary to retinal vein occlusion (RVO), and non-infectious posterior uveitis [36,37]. Its release profile typically includes an early higher-output phase followed by a declining course as the polymer degrades [1,37].

The fluocinolone acetonide implant (Iluvien^®^/Yutiq^®^) is a non-biodegradable microdevice that delivers low-dose corticosteroid exposure (approximately 0.2 μg/day) for up to 36 months, with clinical use in chronic DME and non-infectious posterior uveitis. The near-constant release pattern is accompanied by recognized long-term steroid-related risks, including intraocular pressure elevation and cataract formation [7,38].

The Port Delivery System with ranibizumab (Susvimo) functions as a refillable intravitreal reservoir approximating continuous delivery over several months in nAMD [8]. Its temporary withdrawal and subsequent FDA-approved implant and refill needle updates prior to reintroduction in 2024 underscore the need to integrate durability with device-level safety and ongoing monitoring [9,10].

Experimental platforms—including microspheres, nanoparticles, thermo-responsive hydrogels, and encapsulated-cell systems—aim to extend delivery of anti-VEGF agents, corticosteroids, and neuroprotective proteins. Despite smoother exposure profiles than bolus injections, these technologies remain time-limited or maintenance-dependent strategies because drug output declines as reservoirs deplete or matrices degrade [1,3].

### Interindividual variability and pharmacokinetic challenges

Interindividual variability is a major determinant of ocular pharmacokinetics and therapeutic outcomes [1,3]. Differences in axial length, vitreous volume, and vitreous liquefaction influence the diffusion and clearance of intravitreal agents. Highly myopic eyes may show faster clearance and lower peak concentrations, while other anatomical conditions may prolong drug retention [39,40].

Disease-related changes also modulate exposure. Breakdown of the blood–retina barrier, altered retinal or choroidal permeability, and RPE dysfunction can influence posterior distribution, particularly for large biologics [1,3]. Prior ocular procedures, especially vitrectomy, may accelerate intraocular clearance and shorten the apparent duration of anti-VEGF therapies, as suggested by preclinical and clinical observations [4,41].

These factors help explain why patients receiving similar regimens may demonstrate heterogeneous anatomical and functional responses [1,3]. The resulting clinical variability supports the use of population-based and imaging-informed modeling approaches that incorporate anatomical, procedural, and disease-specific covariates [31,39].

### Limitations of conventional pharmacokinetic models

Traditional ocular PK models often apply simplified one- or two-compartment assumptions with homogeneous distribution and linear elimination [3,31]. Such frameworks may underrepresent concentration gradients between vitreous, retina, and choroid and overlook processes relevant to biologics, including target-mediated binding and tissue-specific uptake, and local degradation [3,31,32].

Even when applied to sustained-release systems, conventional approaches frequently assume static parameters despite disease-driven changes in permeability, inflammation, or tissue remodeling. These limitations are accentuated in chronic retinal disorders requiring long-term exposure stability and in degenerative conditions where progressive cellular loss alters targets and local microenvironment [1,3].

## Pharmacokinetics of gene-based ocular therapy

### From drug dosing to endogenous expression

**Figure 2:**
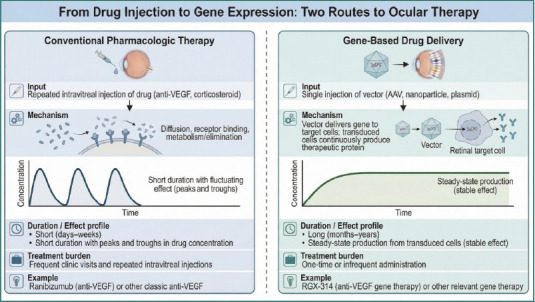
Comparison of conventional pharmacologic therapy and gene-based drug delivery for retinal disease. Conventional therapy typically requires repeated intravitreal injections (e.g., anti-VEGF agents or corticosteroids), producing fluctuating intraocular drug levels with peaks and troughs and a short duration of effect (days to weeks), leading to frequent clinic visits. Gene-based delivery uses a single or infrequent administration of a vector (e.g., AAV, nanoparticle, or plasmid) to transduce retinal target cells, enabling continuous therapeutic protein production and a more stable, long-lasting effect (months to years), thereby reducing treatment burden. Examples shown include ranibizumab (anti-VEGF) and RGX-314 (anti-VEGF gene therapy).

Gene therapy introduces a qualitatively distinct mode of intraocular exposure by enabling endogenous production of therapeutic proteins within ocular tissues [3,11]. Rather than repeatedly administering pharmacologically active molecules that undergo conventional absorption, distribution, metabolism, and elimination, gene-based approaches deliver genetic instructions that allow retinal or RPE cells to synthesize therapeutic proteins over extended periods (Figure 2) [3,11,13]. Once transduction is established, targeted cells function as localized, tissue-confined sources of biologically active agents within the retinal microenvironment [3,42]. This shifts the exposure paradigm from episodic bolus dosing toward continuous, biologically regulated production, fundamentally altering the temporal structure of intraocular drug exposure [31,42].

The resulting concentration-time profiles differ markedly from classical intravitreal PK [32]. Conventional therapies typically produce a rapid post-injection peak followed by a first-order exponential decline, yielding cyclic peaks and troughs that necessitate repeated administration [43,44]. In contrast, gene therapy is characterized by a gradual post-delivery rise in intraocular protein levels, often followed by a sustained plateau and, in some cases, a slow decline over time [26,42]. This profile reflects the sequential biology of vector uptake, transgene transcription and translation, protein secretion, and eventual degradation or clearance [3,26,42].

### Vector biodistribution and expression kinetics

The effective 'input function' of ocular gene therapy is determined by vector design, route of administration, and cellular tropism [3,12]. AAV vectors remain the dominant platform in ophthalmology owing to their favorable safety profile, episomal persistence, and ability to support long-term expression in post-mitotic retinal cells [11,14]. Advances in capsid engineering have expanded retinal transduction efficiency and enabled modulation of cell-type targeting, particularly for delivery routes that avoid surgical retinal detachment [12,14,45]. As a result, vector design has become a central determinant of both the magnitude and durability of gene-derived protein exposure [12,45].

Route of administration remains a primary driver of biodistribution [1,3]. Subretinal injection enables direct access to photoreceptors and RPE, supporting high local expression and forming the foundation of many inherited retinal disease programs [1,14,15]. Intravitreal delivery offers a less invasive, clinic-based approach but is constrained by anatomical barriers such as the inner limiting membrane [1,46]. Engineered capsids, including AAV2.7m8, were developed to enhance retinal transduction from the vitreous [45,46]. Suprachoroidal delivery represents an emerging alternative that may favor chorioretinal exposure while limiting anterior segment distribution [47,48].

Promoter selection further shapes expression magnitude and spatial localization. Strong constitutive promoters can drive higher protein output but may increase the risk of immune recognition or long-term transcriptional attenuation [49,50]. Cell-type–restricted promoters support spatially constrained expression and may improve physiologic alignment and safety, particularly in inherited retinal disorders [12,49].

Collectively, capsid properties, promoter architecture, and route-specific transduction patterns generate a characteristic temporal expression sequence comprising an initial lag phase, a sustained plateau, and, in some cases, a gradual decline [12,26,42]. This biologically regulated sequence contrasts with the diffusion-dominated kinetics of conventional intravitreal drugs [31,43].

### Clinical examples and observed kinetic profiles

Clinical experience provides direct validation of these gene therapy–specific kinetic principles (Table 3) [15,51]. Voretigene neparvovec established proof of concept for durable ocular gene expression through subretinal AAV2 delivery of RPE65 [15,16]. Long-term follow-up and post-authorization data have confirmed sustained functional benefit in a substantial proportion of treated patients. These data demonstrate that a single gene-transfer event can yield multi-year therapeutic activity within the ocular compartment [51-53].

**Table 3:** Selected ocular gene therapy programs in clinical development for retinal diseases

Sponsor / product	Indication(s)	Delivery	Vector	Protein	Status	Key trials
Adverum Biotechnologies – ixoberogene soroparvovec (ADVM022, ixovec)	nAMD	Intravitreal	AAV.7m8	aflibercept	Phase 2 Phase 2 discontinued (safety)	NCT05536973
	DME					NCT04418427
AbbVie / REGENXBIO – ABBVRGX314	nAMD	Subretinal (nAMD) Suprachoroidal (nAMD, DR)	AAV8 (NAV^®^)	antiVEGF monoclonal antibody fragment	Phase 2b/3 Phase 3 Phase 2 Phase 2	NCT04704921 NCT05407636 NCT04514653 NCT04567550
	DR±CI-DME					
4D Molecular Therapeutics – 4D150	nAMD	Intravitreal	Engineered AAV (R100)	dual transgene – afliberceptlike VEGF trap + miRNA targeting VEGFC	Phase 1/2 Phase 2	NCT05197270 NCT05393284
	DME					
Janssen (exHemera) – JNJ81201887 (AAVCAGsCD59HMR59)	GA secondary to AMD	Intravitreal	AAV2	soluble CD59	Phase 2b longterm extension	NCT05811351 NCT06635148
Gyroscope Therapeutics / Novartis – GT005 (PPY988)	GA secondary to AMD	Subretinal	AAV2	complement factor I	Phase 1/2 and Phase 2 discontinued (efficacy) longterm safety followup ongoing	NCT03846193 NCT04437368 NCT04566445 NCT05481827
Avalanche Biotechnologies / Adverum – rAAV.sFLT1 (AVA101)	nAMD	Subretinal	rAAV2	soluble Flt1	Phase 1 and Phase 2a completed with long-term follow-up	NCT01494805
Oxford BioMedica – RetinoStat	nAMD (advanced)	Subretinal	EIAVbased lentiviral vector	endostatin + angiostatin	Phase 1 completed inactive program	NCT01301443
Sanofi Genzyme – AAV2sFLT01	nAMD (advanced)	Intravitreal	AAV2	sFLT01 fusion protein	Phase 1 completed inactive program	NCT01024998

nAMD, neovascular age-related macular degeneration; DME, diabetic macular edema; DR, diabetic retinopathy; CI-DME, center-involved diabetic macular edema; GA, geographic atrophy; AAV, adeno-associated virus; AAV2, adeno-associated virus serotype 2; AAV8, adeno-associated virus serotype 8; AAV.7m8, engineered AAV capsid variant optimized for intravitreal retinal transduction; EIAV, equine infectious anemia virus (lentiviral vector platform); VEGF, vascular endothelial growth factor; anti-VEGF, agent that inhibits VEGF signaling; sFLT-1, soluble Fms-like tyrosine kinase-1 (soluble form of VEGF receptor-1 acting as a decoy receptor); FLT-1 (VEGFR-1), Fms-like tyrosine kinase-1, vascular endothelial growth factor receptor-1; miRNA, microRNA (small non-coding RNA involved in post-transcriptional gene regulation)

Anti-VEGF gene therapy programs extend this paradigm to chronic neovascular disease [17,54]. ABBV-RGX-314 has demonstrated durability signals across early and mid-stage studies and has advanced into global pivotal Phase 3 trials [55,56]. Ixoberogene soroparvovec employs an engineered intravitreal AAV2.7m8 capsid to deliver an aflibercept-encoding transgene [46,57]. Early clinical data indicate prolonged intraocular activity with reduced injection burden in many participants, supporting advancement into pivotal Phase 3 evaluation [20,57].

Not all programs have successfully translated sustained expression into clinical benefit [57]. GT005, an AAV2-based complement factor I gene therapy for geographic atrophy, was discontinued in 2023 [58,59]. This outcome illustrates that durable expression alone may be insufficient for efficacy in complex multifactorial diseases [57,59].

Overall, these clinical trajectories are consistent with exposure patterns characterized by delayed onset followed by extended target engagement [17,26,42]. Where intraocular protein measurements are available, they support persistent presence of gene-derived therapeutic proteins over prolonged intervals [26,57]. These observations align with a quasi–steady-state exposure model rather than bolus decay kinetics [31,43].

### Variability and emerging frameworks in gene therapy pharmacokinetics

As with conventional ocular therapeutics, interindividual variability influences both the magnitude and persistence of gene-derived protein exposure [3,12]. Dose–response relationships are nonlinear and shaped by receptor availability, intracellular processing, route-dependent biodistribution, and vector genome fate [12,60]. Immune activation, corticosteroid prophylaxis strategies, and pre-existing anti-capsid antibodies can further modulate expression kinetics [50,61]. Over longer time horizons, epigenetic regulation and promoter silencing may attenuate transgene output in some contexts [49,62].

Because ocular gene therapy functions as a cell-mediated, biologically regulated system, traditional PK metrics such as *C*_max_ terminal half-life may inadequately describe durability [31,42]. An integrative framework is therefore required that links molecular determinants to measurable intraocular protein concentrations and downstream pharmacodynamic effects [26,31,60].

In this review, the term *genokinetics* refers specifically to the intraocular concentration-time behavior of gene-derived therapeutic proteins [31]. As tunable and potentially reversible expression systems advance, quantitative genokinetic modeling will be essential for improving predictability, managing variability, and defining safety thresholds [60,63].

## Modeling the ocular gene therapy biofactory: mathematical and pharmacologic perspectives

### The ocular biofactory as a pharmacologic system

After successful AAV transduction, ocular cells can sustain local production of therapeutic proteins within anatomically defined compartments [3,11,14]. Depending on the route of administration, expression may be concentrated in the subretinal space and the RPE–photoreceptor interface following subretinal delivery, or extend toward inner retinal territories after intravitreal (and potentially suprachoroidal) administration, reflecting route-dependent biodistribution constraints [12,14,45,47]. This shift changes the primary determinant of intraocular exposure from externally imposed dosing schedules to biologically regulated synthesis and turnover of gene-derived proteins [3,11,26].

From a pharmacologic standpoint, long-term intraocular concentration of a gene-derived product is governed by the balance between effective protein input and protein loss through degradation and/or clearance [3,26,64]. This relationship is conceptually analogous to steady-state conditions in sustained-input systems such as constant-rate infusion [65,66]. However, it differs fundamentally from device-based delivery because the effective 'source term' is cellular rather than mechanical and depends on transduction efficiency, promoter activity, and the abundance and functional integrity of target cells [11,14,26].

### Mathematical representation of expression kinetics

A practical quantitative description of gene-derived intraocular protein exposure can be formulated using an infusion-equivalent pharmacokinetic framework that treats sustained expression as a continuous input rather than episodic bolus dosing [3,12,14]. In this representation, the intraocular concentration of the therapeutic transgene product, *C(t)*, is defined in an effective posterior ocular compartment intended to represent the pharmacologically relevant retina/RPE–subretinal environment, rather than a strictly anatomical fluid space [3,12,14].

Under the assumptions of approximately constant effective protein production and first-order loss, the temporal evolution of *C(t)*, can be described by a single-compartment linear differential equation [3,12,26]:


$$\frac{dC\left(t\right)}{dt}=k_{\text{expr}}-k_{\deg}\cdot C\left(t\right)$$

Here, *k*_expr_ is the effective zero-order input rate in concentration units per unit time (e.g., ng/mL/day) and *k*_deg_ is a first-order loss rate constant (*time*^-1^) capturing degradation and/or clearance from the modeled compartment [3,26,67]. Biologically, *k*_expr_ is an aggregate parameter that subsumes vector uptake and processing, promoter strength, transcription/translation, secretion, and the abundance and functional state of transduced cells [12,14,50]. When expression is conceptualized as an absolute production

rate *R*_expr_ (mass/time), the conversion $k_{\text{expr}}=\frac{R_{\text{expr}}}{v}$ applies, where *V* is the effective modeled compartment volume [3,26]. Solving the equation yields the concentration–time profile [26,67]:


$$C\left(t\right)=\frac{k_{\text{expr}}}{k_{\deg}}\left(1-e^{-k_{\deg}t}\right)+C\left(0\right)e^{-k_{\deg}t}$$

where C(0) is the concentration at baseline (*t*=0).

The corresponding steady-state (plateau) concentration is therefore $C_{ss}=\frac{k_{\text{expr}}}{k_{\deg}}$ providing a direct quantitative link between effective production and loss [26,67].

The time required to approach steady state depends only on *k*_deg_, and the time to reach a fraction *p* of steady state is *t*_p_=-ln(1-p)/*k*_deg_ [66,67]. Using standard pharmacokinetic relationships, the half-life associated with first-order loss is $t_{1/2}=\frac{0.693}{k_{\deg}}$, implying that ~2.3, ~3.0, and ~3.5 half-lives correspond to ~90%, ~95%, and ~97% of steady state, respectively [66,67].

In ocular gene therapy, a biologically meaningful lag phase often precedes detectable protein expression due to vector internalization, intracellular trafficking, and transcriptional activation, and this behavior can be represented by introducing a lag time *t*_lag_ without changing the steady-state relationship [12,14,50]. This single-compartment infusion-equivalent model is intended as a transparent starting point for interpreting gene-derived exposure and does not capture spatial gradients or exchange among retina, vitreous, and aqueous humor [3,26,31].

When bridging posterior expression to clinically feasible sampling matrices (e.g., aqueous humor) or when spatial heterogeneity is central, multi-compartment or spatially informed ocular PK models are more appropriate [3,26,31].

### Concept of genokinetics

Traditional pharmacokinetics describes the disposition of exogenous drugs using absorption, distribution, metabolism, and elimination [3,31]. Gene therapy follows a distinct sequence of rate-controlling steps—vector entry, intracellular processing, transcription, translation, secretion, and protein turnover—which together determine the intraocular concentration–time behavior of the therapeutic transgene product [11,14].

In this review, we use genokinetics narrowly to denote the intraocular exposure profile of gene-derived therapeutic proteins, rather than systemic vector biodistribution [11,26]. Within this operational scope, parameters such as *k*_deg_, *k*_expr_, *C*_ss_ and *t*_ss_ provide a quantitative interface between molecular expression biology and established pharmacokinetics–pharmacodynamics (PK–PD) reasoning [26,31]. This framing supports the application of compartmental methods and population approaches to interpret durability signals, quantify interindividual variability, and refine exposure-related safety thresholds in late-stage ocular gene therapy programs [26,60].

### Model limitations

Although infusion-equivalent modeling provides an interpretable abstraction for gene-derived intraocular exposure, predictive accuracy is constrained by ocular spatial heterogeneity and compartment-specific clearance, which can generate gradients between subretinal tissues, retina, vitreous, and aqueous humor and may require multi-compartment or spatial models when bridging to clinically accessible sampling matrices [3,26,31].

Expression is also not guaranteed to scale linearly with vector dose, as effective production may plateau due to saturable cellular uptake/processing, limits in transducible target cell pools, or promoter-related ceilings [11,26].

Key parameters can vary over time: immune activation and inflammation, transcriptional attenuation (including epigenetic mechanisms), and disease progression may reduce effective *k*_expr_, while changes in tissue integrity or proteolysis may alter *k*_deg_, shifting the apparent steady state and contributing to gradual declines in expression in some patients [11,14,26,50].

Interindividual variability—driven by route-dependent biodistribution, ocular anatomy, prior surgery, and immune status—supports a model-informed, covariate-based approach and underscores the importance of long-term safety surveillance and follow-up plans consistent with regulatory expectations for gene therapy products [3,26].

Finally, exposure must be quantitatively linked to clinically meaningful pharmacodynamic endpoints (e.g., OCT biomarkers, rescue treatment frequency, visual function) because durable expression alone does not ensure efficacy across diverse targets [60].

## Pharmacodynamic and clinical implications of gene-based ocular therapy

### PK–PD integration and sustained target engagement

In gene-based ocular therapy, pharmacodynamic effects are directly coupled to the kinetics of transgene-driven protein expression and turnover [3,26,60]. The steady-state intraocular concentration *C*_ss_ achieved by transduced cells is therefore a central determinant of both effect magnitude and durability [26,60]. Unlike conventional intravitreal injections, which produce peak-trough exposure profiles, gene-derived proteins can generate a more stable concentration-time course, supporting continuous target engagement with reduced temporal fluctuation in downstream signaling [3,31].

Within an infusion-equivalent framework, therapeutic efficacy is expected to persist as long as intraocular protein levels remain above a minimal effective concentration [26,60]. This paradigm is most extensively explored in anti-VEGF gene therapy for nAMD [17,56]. Programs such as ABBV-RGX-314 (sura-vec), delivered subretinally or via the suprachoroidal route, and ixoberogene soroparvovec (ixo-vec), delivered intravitreally, have demonstrated sustained intraocular anti-VEGF activity, durable anatomic control, and reduced reliance on rescue injections in early- and mid-stage clinical studies [54-56].

Collectively, these data illustrate how a favorable balance between expression and clearance can translate into prolonged pathway suppression from a single administration [26,60]. From a PK–PD perspective, this supports a shift away from repeated bolus dosing toward a more stable pharmacodynamic environment in responsive patients [3,31,60].

### Beyond angiogenesis: expanding pharmacodynamic horizons

Although anti-VEGF gene therapy currently provides the most advanced clinical validation of sustained gene-derived exposure, similar PK–PD principles are being applied to other retinal disease pathways [26,60]. Complement-modulating gene therapies, such as AAV-based delivery of complement factor I for geographic atrophy, were designed to restore homeostatic regulation of the complement cascade through long-term local expression [57,59]. The discontinuation of GT005 following phase 2 evaluation underscores an important principle: durable expression alone does not guarantee clinical efficacy in multifactorial diseases [16,57].

Beyond complement modulation, anti-inflammatory and neuroprotective strategies are under investigation for conditions such as chronic noninfectious uveitis, inherited retinal degenerations, and glaucoma [14,16]. In these settings, the pharmacodynamic objective is analogous—to maintain a long-term steady state of therapeutic protein sufficient to continuously modulate pathogenic signaling while avoiding overexposure [26,60]. Operationally, this can be expressed as maintaining *C*_ss_ at or above a pathway-specific pharmacodynamic threshold *C*_min,PD_ benefit is unlikely [26,60].

### Safety, regulation, and therapeutic windows

Continuous intraocular expression introduces distinct safety and regulatory considerations [50,63,68,69]. Because gene-derived protein output is biologically maintained rather than externally titratable, excessive or mislocalized expression cannot be rapidly reversed [50,62]. For anti-VEGF constructs, prolonged oversuppression has been discussed as a potential risk to chorioretinal homeostasis, while intravitreal delivery platforms have highlighted dose-dependent inflammatory responses, emphasizing the importance of careful dose selection and monitoring [49,55,56].

Immune responses to AAV capsids or transgene products, as well as pathway-specific consequences of chronic modulation, further reinforce the need to define ocular therapeutic windows for gene-derived proteins [14,50,62]. Conceptually, optimal gene-based therapies should achieve a stable *C*_ss_ that exceeds efficacy thresholds while remaining below toxicity limits [26,60].

Emerging strategies in synthetic biology—including inducible or ligand-responsive promoters and tunable expression cassettes—aim to provide post-administration control over intraocular protein output [70,71]. As these technologies mature, integration of predictive PK–PD modeling with imaging and fluid biomarkers will be critical to preserving durable efficacy while maintaining long-term retinal homeostasis [26,50,60,68,69].

## Future directions and perspectives in gene-based ocular pharmacotherapy

Future advances in ocular gene therapy will increasingly depend on the integration of quantitative pharmacokinetic modeling, vector engineering, and clinically informed data analytics [26,60]. Development is moving beyond static expression constructs toward platforms designed to improve predictability of expression magnitude and durability, enhance safety, and enable greater flexibility after administration [15,60].

Synthetic biology approaches—such as inducible promoters, ligand-responsive regulatory elements, and cell-type–specific expression architectures—offer potential solutions for dynamically adjusting intraocular protein output in response to disease activity or emerging safety signals [70]. These systems may expand the therapeutic window by allowing sustained pathway modulation when beneficial, while mitigating risks associated with prolonged overexposure [70].

In parallel, model-informed and data-driven strategies are expected to strengthen personalization of gene-based therapy [26,60]. Integrating imaging biomarkers, patient-specific ocular characteristics, and immune-related factors into predictive genokinetic–pharmacodynamic frameworks may improve estimation of steady-state exposure and durability prior to treatment [26,60,56]. Such approaches could support rational patient selection and dose optimization across diverse retinal conditions [56,60].

## Conclusion

Gene-based ocular therapy represents a paradigm shift from repeated exogenous dosing toward sustained intraocular production of therapeutic proteins. By enabling long-term expression within targeted retinal cells, these approaches generate exposure patterns fundamentally distinct from conventional intravitreal pharmacokinetics and hold the potential to reduce treatment burden in chronic retinal disease.

This review integrates classical pharmacokinetic reasoning with molecular determinants of transgene expression to frame ocular gene therapy as a biologically regulated sustained-delivery system. Continued progress will depend on refining quantitative models of expression and turnover, strengthening PK–PD linkage to clinically meaningful biomarkers, and advancing expression-control strategies that maintain exposure within safe and effective therapeutic windows. As these components evolve, gene-based delivery may become an increasingly predictable and customizable element of long-term retinal pharmacotherapy.
